# Transactions of the Medical and Physical Society of Calcutta, Vol. 1

**Published:** 1826-07

**Authors:** 


*Transactions of the Medical and Physical Society of Calcutta.* Vol. the first. 8vo. pp. 410. Calcutta, 1825.

WE have often deplored the situation of our medical brethren in the vast regions of the East, cut off, as they are, from that speedy intellectual intercourse which forms one of the chief enjoyments of professional men in Europe and America—and counterbalances much of the anxiety, and toil, and crosses, which every medical man is destined to experience, more or less, in the discharge of his arduous duties. The great majority of the profession in India are scattered far apart, over a vast extent of country. They hear and see nothing of the stir of science, and catch but indistinct and partial glimpses of the advancement of knowledge.

"Their limited means and frequent removals, put it out of their power to provide themselves with regular and expensive supplies of books, and there are no public libraries beyond the limits of the Presidencies: they can derive, therefore, little benefit from the recorded experience of others, and they have rarely any opportunity of confirming or correcting their own, by that of an associate, a rival, or a friend:—their scene of action is too restricted, and too entirely their own, to admit of emulation or ambition; and the official reports, in which alone their practice is commemorated, are too much matters of form and routine, to merit or attract animadversion. So situated, it is not wonderful that their zeal dies within them: no man is independent of external stimulus, and the surest method of annihilating energy, is to leave it to prey upon itself." vi.

We trust that we have, in some degree, with our cotemporaries, contributed to lessen these evils, by putting it in the power of every individual of the profession in the East, as well as in the West, to see a concentrated exposé of all that is going on in the medical world, at a very small expense, and in a form which the soldier in the field might carry without inconvenience. Nevertheless, there was something more than the journals of Europe wanted. A medical society was desirable, and was accordingly formed at Calcutta, under favourable auspices, enrolling a great number of names, and including men of rank, talent, and science in all of the three Presidencies. The main object of this society was to give a concentric impulse to the detached members of the service, and to afford them augmented facility of information, as well as a new excitement to emulative exertion. They were invited to contribute without restraint, the results of their enquiries and observations, while, on the other hand, the proceedings of the meetings at the Presidency were regularly communicated to the non-resident members. The result of the appeal fully justified expectation, and proved that means only were wanting to animate the energies of the service. Publication was only a possible contingency, at first; but various contributions on professional subjects rapidly accumulated, and soon put the matter out of doubt. The volume before us is the first fruit of the society, and, all things considered, does credit to the new association, and gives promise of still better things in future.

This volume consists of thirty-three original articles, and an appendix of minor matters, consisting of intelligence, extracts of letters, regulations of the society, &c. It cannot be expected that all these articles should be equally interesting to the European reader. Some of them are of much more local than general importance, as may be readily supposed from the nature of the circumstances under which the work is published. This feature, however, will rather augment than decrease its value in the East, where, of course, the principal scene of its circulation and success must lie.

I. The first article is a very erudite one on the Kushta, or leprosy, as known to the Hindoos, from the pen of H. H. Wilson, Esq. It will be read with much interest by our Oriental brethren, and to them we must consign it.

II. The second article is by Dr. N. Wallick, giving a description of the tree which produces the Nepal camphor wood, and sassafras bark. This tree was discovered by our author on his journey to Nepal, and a drawing of it, which he designates by the name of Laurus Glandulifera, is given.

III. *Fatal bite of a Venomous Snake.* By P. Breton, Esq.—On the 27th March, 1818, a Syce was bit in the leg by a snake, called by the natives *siah chanda.* A piece of string had been immediately tied very tight above the part, and the absorption of the virus seemed to have been retarded. Half an hour after the accident, the Syce presented himself to Mr. Breton, and evinced no symptoms of disorder. Two punctured wounds, an inch apart, were seen near the ankle, from which a few drops of blood had issued. No discoloration or swelling had taken place, nor any sense of pain in the part. Mr. B. however, applied the caustic volatile alkali, and ordered some of it to be administered internally. In a few minutes after this, the Syce became sick, began to vomit, and complained of faintness and indescribable sensations of uneasiness. Fifteen drops more of the volatile alkali were immediately given, and the dose was quickly followed by vomiting. There was now a considerable diminution of the force of the circulation. Vomiting again recurred, after which there was a suspension of the circulation, loss of sense, and convulsions. Some more of the volatile alkali seemed to rouse the circulation and check the convulsion; but the pulse soon fell again—the body became cold—respiration suspended—and the vital powers appeared, for a few seconds, extinct. 20 drops of the volatile alkali were forced down the throat, when a convulsive effort was made to breathe, and a tremulous motion of the heart and arteries followed. As the circulation returned, so did the senses—the faintness, dimness of sight, and sickness continued. Some more volatile alkali being given, the man seemed gradually to recover from the effects of the poison, and remained tolerably well during the night. In the morning the leg and thigh were found much swelled—he was perfectly sensible, but complained of pain in the limb, dimness of sight, faintness, and restlessness. The affected limb was quite cold and tense to the feel—the pulse imperceptible—the tongue colourless. Some more volatile alkali was administered, and repeated every ten minutes. About ten o'clock a slight convulsion occurred, and soon put an end to his existence.

*Remarks.* As the effects of the poison did not manifest themselves till an hour after the bite, it is more than probable that, had cupping-glasses been employed, in the manner recommended by Dr. Barry, this man's life might have been saved. We strongly recommend our brethren to take every opportunity of putting in force the ingenious and easy method of prevention so successfully experimented on by Dr. Barry.

IV. *Wound of the Stomach.* Mr. Breton communicates this case also. The patient was a trooper in the irregular cavalry, who, in a fit of despondency, charged his holster pistol with three balls and attempted to destroy himself. His native woman, however, flew to him and endeavoured to wrest the pistol from his hand, and for this generous action forfeited her own life, for the pistol went off in the struggle, and the contents went through the woman's body, who died on the spot! The trooper immediately re-loaded, and shot himself in the pit of the stomach, the ball lodging in the left side of the lumbar vertebræ. He had no more ball cartridge left, and, although he fell, from the first shock, yet he was able to walk to the fort soon afterwards, where he related the whole tragedy.

"On examining his wound, I found that the ball had entered the epigastric region, and was perceptible to the feel in the left side of the loins. I immediately extracted it, and closed the incision with adhesive plaster. Conceiving that the stomach was penetrated by the ball, I considered the case a hopeless one, and reported the wound to be mortal. The following morning, the man had recovered his senses, so as to give a distinct and rational account of all the circumstances which led to the commission of the rash act; and the wound manifested such appearances, as left no doubt in my mind of the stomach being perforated.

"My attention was therefore directed to uniting the anterior wound with all possible speed, and to prevent the introduction into the stomach of any more food than was absolutely necessary to support life. To my astonishment, the wound quickly united, and the serous discharge from the stomach gradually ceased. When union had taken place in the anterior wound, I was informed by the native doctor, that when food was taken into the stomach, particles now and then escaped from the wound in the loins, where the ball lodged. To convince myself of the truth of this assertion, I gave the trooper a draught of water to drink; and almost immediately afterwards, I distinctly saw a small quantity of it trickle through the wound. I repeated this two or three times, and was satisfied that there was a communication between the stomach and the wound in the loins. Superficial dressings were continued, and rigid abstinence enjoined. In the course of about a month and a half, the wounds healed, and the man recovered, and was afterwards tried by a General Court Martial for the crime of murder." 61.

Mr. Breton refers to Mr. King, surgeon of the 4th Native Regiment for corroboration of the foregoing particulars. We are sorry Mr. B. did not give a more circumstantial account of the appearances manifested by the wound on the morning after the accident, and which, he says, left no doubt in his mind respecting the perforation of the stomach.

V. *Oil in the Blood.* This is an unusual phenomenon, but there are several instances on record. Dr. Adam relates the case of a Sergeant M'Donald, who went to bed in good health, though somewhat intoxicated, and was found dead in the morning. As suspicions were entertained of some foul means being used, the body was carefully examined. There were no marks of violence internal or external; but the vessels of the brain were found rather distended; both there and in other parts of the body, there was distinctly seen a quantity of oil floating in the blood.

VI. *Delirium Tremens.* Mr. G. Playfair has seen a considerable number of cases of this curious disease; and, therefore, has furnished the Society with a paper on the subject. It happened only among those men addicted to habitual excess in the use of spirituous liquors. In some instances it assumed the form of outrageous mania, and if neglected or improperly treated, the worst consequences were to be apprehended.

"The subjects above alluded to are sent into hospital from the immediate effects of the debauch, viz. extreme irritability, tremors, sensation of debility, flushed countenance, eyes heavy, severe head-ache, (though this is not an invariable symptom,) pains and spasms in the stomach and bowels, constipation, incessant vomiting, and frequent violent spasms all over the body and extremities. To remove this state of the system, the usual means resorted to are—

"1st. To allay the inordinate action of the diaphragm and abdominal muscles, and general irritability of the nervous system, by the exhibition of saline draughts in a state of effervescence, camphorated mixture, laudanum, ether, blood-letting, general and local, blisters, sinapisms, &c.

"2nd. A full dose of calomel, viz. gr. xx. with or without laudanum, as a sedative, and preparatory to,

"3rd. Strong purgatives and enemata, repeated as may be found necessary.

"This treatment is generally successful in removing the effects of the debauch, in the course of one or two days, after which, recovery to health and strength is usually rapidly progressive; but it sometimes happens, that, from the time of the admission of the patient, there prevails a great degree of restlessness, and want of sleep, which are always to be looked upon with suspicion, as the forerunners of something more serious; and the cure is never to be considered complete, till the patient shall have had some hours sound repose: for this purpose anodyne draughts are liberally exhibited, the instant the bowels are freely evacuated." 126.

In some instances, however, the restlessness continues, with a particular wildness in the eye, and a dislike or want of power to remain quiet. These symptoms gradually increase—he cannot lie down—or, if he does, he starts up as if likely to be suffocated. He talks in a strange manner, yet with the appearance of being perfectly in his senses—complains of the other patients making faces at him, and entering into conspiracies against him, &c. &c. This is accompanied, of course, by want of sleep. It now becomes necessary to separate the patient from others, to prevent mischief—and restraint itself is soon indispensable. Our author's experience does not coincide with those who believe that the disease only takes place in advanced life—on the contrary, he has found it almost invariably affect the young, the religious, the robust, the plethoric, and especially those who have been suddenly debilitated by previous illness. The mode of treatment in this case is to procure sleep, as this has always relieved the complaint. With this view, a full anodyne draught was exhibited at bed-time, and sometimes repeated at midnight, if not efficient by that time. When symptoms of mental derangement took place, he commenced with, perhaps, 100 drops of tincture of hyoscyamus, and 30 of laudanum, repeating 20 of the former, and 10 of the latter, every two hours, as regularly as possible till sleep was procured. He has succeeded in twelve hours, but it has required 24, and even 36 hours before irritation could be allayed, or sleep procured. He has been obliged to suspend the use of opium, till the bowels were well evacuated, but he generally continued the use of henbane. On several occasions he has been obliged to have recourse to the lancet, alarmed by the fulness of the pulse, inflamed appearance of the eyes, and obstinate constipation of the bowels. After the bleeding, opiates had a better effect. Tremors were not always present—the patient seldom complained of head-ache—the pulse was generally regular, and rather full—yet he has seen it feeble, and nearly imperceptible, the body being cold, and the sweat clammy. In these cases he has resorted to wine and other stimulants. Patients of this kind are almost always bathed in sweat—the tongue usually clean and moist, with great thirst. No case, in our author's practice, proved fatal; and in this, we think, he was very fortunate, for it is a dangerous disease.

VII. *Calculus Adherent.* Mr. Corbyn relates this case. A man complained of most agonising pain, from obstruction in the urethra, impeding the discharge of urine, which passed guttatim. The urine was peculiarly offensive, and impregnated with calcareous and viscous matter. There was tension and pain over and above the symphysis pubis. The pulse was low and tremulous—the bowels obstructed—countenance haggard. He was also under sentence of punishment, which added greatly to his sufferings. On passing a sound into the urethra, one or two small abscesses were ruptured, and some matter escaped. Mr. C. could then distinctly perceive a large stone in the bladder. Light diet, laxatives, and laudanum. The symptoms became daily more urgent and dangerous, and dissolution was threatened, unless lithotomy was performed. An hour was appointed, but such bad symptoms came on that the operation was obliged to be deferred, and the patient soon died. On dissection, the urethra was observed to be much diseased, and the stone was found so adherent to the coats of the bladder, that it could not be disentangled without tearing the organ. The bladder was, in fact, a mass of disease. Nothing but the high operation could have been of any service here.

VIII. *Pedicular Eruptive Disease.* About a month previous to December 6, 1823, Mr. Sinclair was called to a gentleman in the province of Candeish, labouring under those symptoms which generally usher in fever, attended by much depression of spirits. This state continued until the evening of the third day, when the pain in the head became more severe, and the face unusually flushed. Bleeding was resolved on, which instantly relieved all the symptoms. In less than half an hour he complained of great itching all over him—about midnight this feeling had become intolerable, and at eight o'clock the next morning, on examining his skin, he discovered an eruption resembling the prickly heat, but more in clusters, and the pimples of larger size, and open at the top.

" Around these were numbers of a small insect: they were to be seen within others, and in the few that had not been opened, the bags of ova were easily extracted with the point of a needle, and were of sufficient size to be distinctly observable by the naked eye. The parts particularly affected were the back, and back part of the neck. I ordered the patient to be slightly rubbed with the Ungt. Hyd. Mit. which in the course of that day relieved him completely, and destroyed the insect." 150.

IX. *Dracunculus.* Mr. Bird has made some observations on this curious disease, and thinks if they are corroborated by future observers, we shall have arrived at a nearer acquaintance with its nature.

" They will at once corroborate the opinion, that it originates in something peculiar to the soil or water of the place, and that this peculiarity is, their containing some animal whose ovum, when deposited in the human body, is capable of producing a creature sui generis." 154.

Mr. Bird does not think, however, that dracunculi are produced from the ova of an insect " something after the manner of the chigre of the West Indies."

" The great objection to this opinion seems to be, that the length of the dracunculus is out of all proportion to the size of the larva of any known insect, and that its structure more closely resembles the vermes than any other class known to naturalists." 155.

Leaving speculation aside, we shall give the following therapeutical extract from our author.

"When the body of the Dracunculus is of sufficient firmness to allow of its being pulled, the common method of rolling the animal on a quilled piece of adhesive plaster, and thus extracting it by degrees, will be found sufficiently successful to justify its being generally adopted, in opposition to the plan recommended by M. Larrey, of snipping off the end of the worm close to the opening in the skin, and then promoting suppuration by fomentation and poultices. It is, however, frequently rendered abortive by the ill-timed attempts, which patients sometimes make to extract the worm. When the surrounding parts are tense and inflamed, any effort made to draw the Dracunculus from its bed generally breaks it; and tedious suppurations follow in consequence. Under such circumstances, I have always waited until pus was formed round the body of the animal, and a serous ulcer had been in this manner established. Fomentations of warm water, and friction with oil, have been the only means I have ever employed to obtain this result. Leeches are often objectionable, as the inflammation is of the anasarcous kind, and they are apt to produce an increase of the swelling, and troublesome ulcerations. When they are deemed advisable, they should be applied, I think, in the neighbourhood of the swelled parts. A piece of simple dressing over the opening in the skin is preferable to a poultice; for under the use of the latter, I have always seen the Dracunculus drop off. In two cases, where the constitution was very irritable, I mercurialized the system, and the effect it had in subduing the inflammation was very remarkable. This practice appeared to kill the worms, which were discharged in bits, along with the pus of the parts. When the body of the Dracunculus is so fine as not to allow its being extracted in the common way, and the inflammation of the parts runs high, mercurializing the system may be adopted, I think, with advantage. When there is swelling, with hardness of the soft parts, without any considerable redness of the skin, friction with mercurial liniment has considerable power in relieving tension, and promoting suppuration." 63.

X. *Aneurism of the Aorta. By Dr. Adam.*—The subject of this curious case was a seaman, aged 32 years, from one of the Company's ships in the river, admitted into hospital on the 9th October, 1822, complaining of severe pain in the right shoulder extending down the inside of the arm to the extremities of the fingers. It was most acute and permanent immediately under the spine of the scapula, reaching outwards to the acromion. Voice feeble, and feels occasional difficulty of breathing. At the lower part of the neck, on the right side, and directly over the clavicle, there is an irregular swelling, the size of a goose's egg, rather firm, reddish, and pulsating. In the brachial and radial arteries of the wrist there is scarcely any pulse. In the opposite side it is full, and 92 in the minute. The carotids beat violently—he is attacked with occasional head-ache, principally in the back part—the heart appears to labour, but no sense of throbbing in the chest. Complains of pain under the false ribs of the right side. Dates the complaint about five months previously, when leaving England, after taking cold, and being affected with much difficulty of breathing.

"From the above symptoms, the nature of the case seemed quite evident; and it was resolved that the patient should be very freely blooded, and put upon the strictest antiphlogistic regimen in the first instance. Forty ounces of blood were accordingly drawn from the arm at two bleedings, and he was purged by means of sulphate of magnesia daily administered. On the 12th, there was not as formerly any pain in the tumour itself; but it was equally severe in the shoulder and down the arm, and the whole limb was in a state of numbness, or, as he expressed it, felt, "like a lump of lead." Sleep very much disturbed. On the 13th, he had enjoyed some repose during the night, and the tumour was not only sensibly diminished, but there also appeared some degree of improvement in his voice; the pain continuing much the same. Anodyne liniments were gently rubbed in over the shoulder and arm, (consisting of camphorated tincture of soap and laudanum,) and opiates, with Dhatura extract, (one third grain every second hour,) were exhibited internally, and the bowels kept open by means of castor oil. On the 16th, he was again bled to twenty ounces. On the 20th, he complained of severe pain in the belly, and V. S. was again ordered, which he would not submit to. On the 22d, a solution of tartar emetic was prescribed, so as to produce nausea; but vomiting being excited, this remedy was intermitted. On the 26th, Ipecacuanha was administered in the dose of three grains every second hour, and he was bled to oz. xv, which he readily submitted to. The Ipecacuanha was persisted in, so as to keep up a constant nausea, and lower the force of the circulation; and on the 1st Nov. the pulsation in the tumour appeared sensibly weaker. Two days after, a pain attacking him in the back of the head, leeches were applied to the temples (xx,) and he was freely purged at the same time. The pain of the neck continuing, the leeches were repeated, and the Ipecacuanha was still administered with an intermission of a day or two. On the 12th, having complained of pain in the chest, he was again bled to oz. xvi, and the anodyne liniment repeated, which afforded some relief. A considerable elevation of the back was noticed for the first time on the 15th. It was situated to the right of the spine, between that and the scapula, and appeared to depend on some internal organic disease. On the 18th, the pain both in the neck and chest was very severe, and he remarked, that he could *not lie at all on his right side.* Anodyne remedies were then had recourse to, principally Hyoscyamus, administered in the form of extract. From the 23d to the 26th, he complained of feverish symptoms, and the tumour was painful again, accompanied with difficult respiration, cough, and occasional fits of sneezing. By the 29th, the tumour was evidently increased, and his face was swollen, with an aspect of anxiety which it had never exhibited before; and he dreaded suffocation at times. On the 1st December, he was, however, better; and continued improving from that date to the 27th of the same month." 231.

Venesection was resorted to several times afterwards, and ipecacuan was given so as to keep up a tendency to nausea. The most rigid abstinence was, of course, enjoined. The good effects of this treatment were very obvious. By the 8th of December, the tumour had nearly disappeared, and the pulsation was also diminished, while the pulse at the wrist became more developed. This state of improvement continued, with very little alteration till the 6th June, when he was attacked with pains on the opposite side of the shoulder and neck. In the latter end of July, his bowels became affected, and he was twice bled to twenty ounces each time. An obstruction appeared to exist in the intestinal canal, and it required various and copious cathartics to remove this obstruction; but it was at length done. He still complained of pain in the lower part of the right side, in the situation of the colon. He also stated that, some years before, he had been cut for a rupture. *9th August.* A swelling made its appearance over the sternal extremity of the clavicle, the surface slightly reddened, and exceedingly painful on pressure; yet the next morning the swelling was entirely gone off, and he made no complaint, except of his bowels. On the 20th August, however, he expired.

"*Dissection.* The Aneurismal Tumor, although obviously not the immediate cause of death, attracted our attention in the first instance. Externally it was scarcely visible, excepting at the sternal extremity of the clavicle, which appeared in some degree elevated. On dissecting off the integuments, this bone was found unconnected with the sternum—bare at the extremity—and at some points of its surface rough from the effects of incipient absorption. Having removed the clavicle entirely, the tumor itself came into view, in the shape here of a small nipple-like process, rugous on the surface, and of a dark venous blood, or mulberry color. It appeared to be the coagulated blood much compressed, and shrinking into a smaller compass than it formerly occupied. The ribs being raised, and sawn off, the whole extent of the Aneurism was displayed, proceeding from the *Arteria Innominata*, and filling the upper part of the thorax, but altogether not exceeding in size the fist half closed. The jugular vein and the nerves were seen stretched over the surface of the swelling, and the carotid and subclavian arteries were traced to their origin, but found there quite impervious. The carotid had its orifice plugged up by a firm fleshy substance, while the subclavian seemed united by a ligamentous structure. The blood in the *centre* of the tumor was coagulated, like currant jelly; but from this to the circumference it gradually acquired more firmness; and the concentric layers into which it was disposed resembled in color and texture shavings of thick tanned leather, when moistened. The cyst of the aneurism was thin towards the upper part of the tumor; and became thicker towards the artery. That of the nipple-like projecting process was also thin, but shewed no disposition to rupture.

"About an inch lower down, another aneurism was found, projecting from the right side of the *Aorta Ascendens*, in a very regular globose form. It seemed to be of comparatively recent formation, as the blood in it was merely coagulated, without having assumed any of the firm laminated structure described above. It did not exceed the size of a walnut, and communicated with the canal of the vessel by an orifice, which admitted the point of the finger, quite smooth at the edges; and the vessel itself was smoother here, and apparently more sound than at any other part of its internal surface. In general this was rough and unequal, arising from disease in the *muscular coat*, which could be most distinctly felt, when the vessel was grasped between the finger and thumb. The coats, upon the whole, however, seemed thinner than natural, and the calibre of the vessel was enlarged all the way from the heart; which circumstance produced a want of elasticity, and occasioned its lying more flat and flabby-like than we observe in the sound condition of these parts." 237.

An abscess was also found in the liver, containing a quantity of curdy pus. The substance felt soft to the touch, and had obviously undergone suppuration. The whole left lobe had been converted into either pus or serum—nothing remained but a thin cyst-like wall to the viscus. The stomach and spleen were healthy.

We have thus drawn the attention of our brethren to a proportion of the contents of this oriental stranger, and recommend it to the protection of its occidental brethren.

